# Sub-coronary aortic root replacement post-aortic valve dehiscence: a case report

**DOI:** 10.1186/s13019-024-02545-w

**Published:** 2024-03-26

**Authors:** Aabha Divya, Tobin Mangel, Rakesh Seetharaman, John Fouad Taghavi

**Affiliations:** 1 Cardiac Surgery, Royal Papworth Hospital NHS Trust, Cambridge, CB2 0AY UK; 2 Cardiothoracic Intensive Care, Royal Papworth Hospital NHS Trust, Cambridge, UK

**Keywords:** Redo aortic surgery, Anomalous coronary anatomy, Alternate aortic remodelling

## Abstract

This is a case report of a 78-year-old male who underwent a sub-coronary aortic valve and root replacement due to valve dehiscence and aortic root pseudoaneurysm. The patient had complex anomalous coronary anatomy and had undergone a previous tissue aortic valve replacement in 2013. The patient made an uneventful recovery and was discharged from the hospital five days later. The authors suggest that the sub-coronary root replacement technique should be considered in elderly patients and patients with complex coronary anatomy.

## Introduction

We present a case of a 78-year-old male undergoing a sub-coronary aortic valve and root replacement due to valve dehiscence and the presence of aortic root pseudoaneurysm. While aortic valve reoperations are not uncommon, our case is distinguished by the convergence of complex anatomical anomalies involving the coronary arteries and the coexistence of an aortic root pseudoaneurysm.

In this report, we delve into the intricate surgical intervention required to address these multifaceted challenges. By sharing this case, we aim to contribute to the clinical and educational narrative surrounding such complex cardiac procedures. Our experience underscores the critical need for elderly tailored surgical approaches in cases of intricate coronary anatomy, valve pathologies, and aortic root abnormalities.

### Case report

**Fig. 1:**
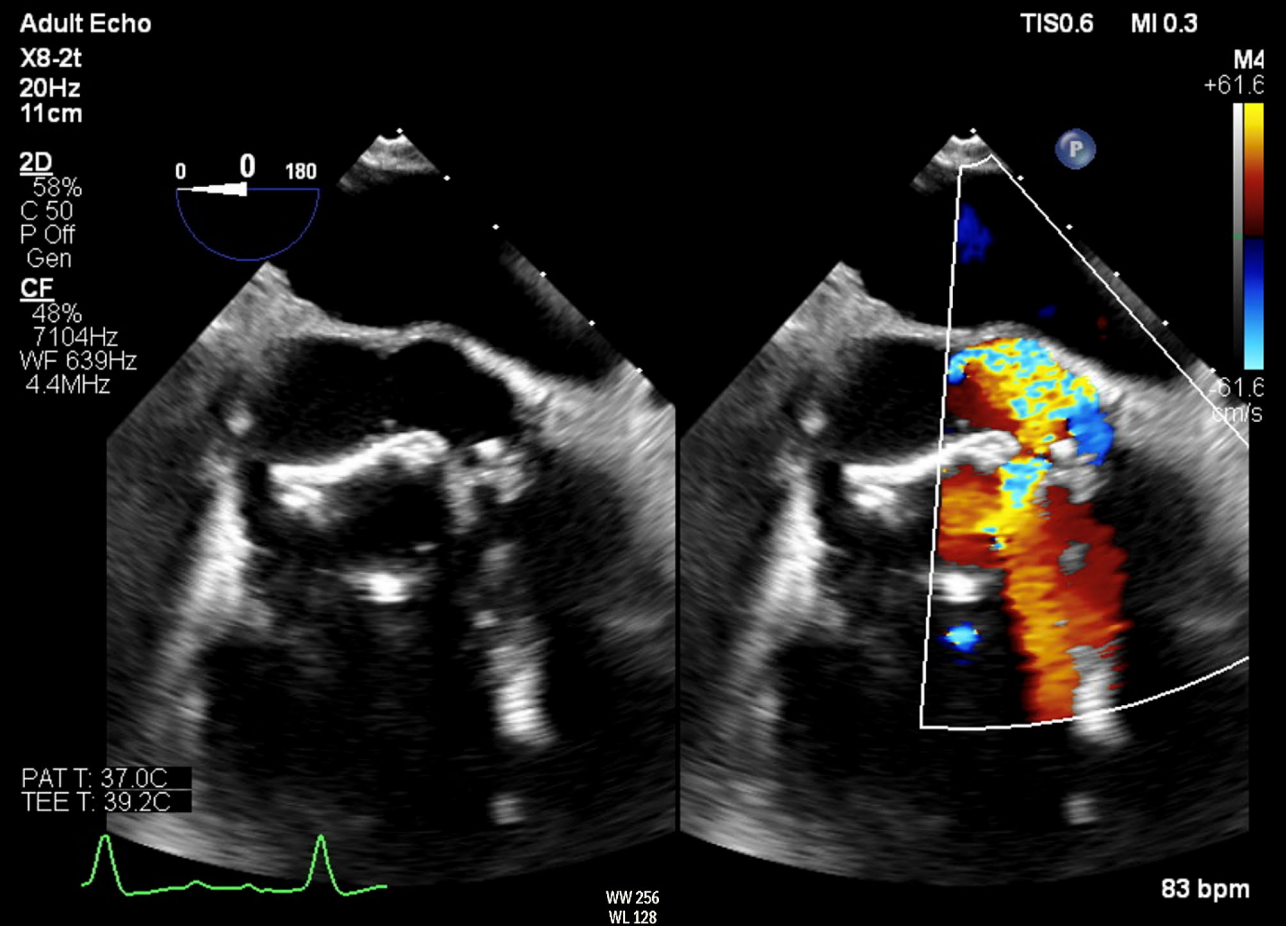
Transoesophageal echocardiogram short axis view of the rocking aortic valve

A 78-year-old male, with a past medical history of type II diabetes, chronic kidney disease, Barrett’s oesophagus and atrial fibrillation, and a previous tissue aortic valve replacement with a 23 mm Mitroflow aortic pericardial valve (Sorin Medical, NY, USA) in 2013, presented to hospital in November 2021 initially with a right parietal stroke which he underwent successful thrombolysis. The patient made a full recovery prior to his admission to our hospital in December 2021. The aetiology of the stroke is unclear but was thought to either be due to chronic atrial fibrillation or as sequalae from prosthetic valve endocarditis. Due to the previous surgery, there was concern of prosthetic valve endocarditis and the patient was started on empiric therapy which included vancomycin and rifampicin followed by daptomycin and rifampicin. He was on antibiotics for six weeks despite having four negative cultures. Repeat cultures were taken post antibiotic therapy all of which returned as negative. Transthoracic and transoesophageal echocardiography demonstrated rocking of the prosthetic valve with dehiscence at the aortomitral continuity level in systole (Fig. 1). Computed tomography pulmonary angiogram (CTPA) demonstrated a possible pseudoaneurysm of the aortic root. The patient was accepted for urgent redo sternotomy, sub-coronary aortic root replacement and exclusion of aortic root pseudoaneurysm within days of referral and was operated two days after admission to our hospital.

**Fig. 2:**
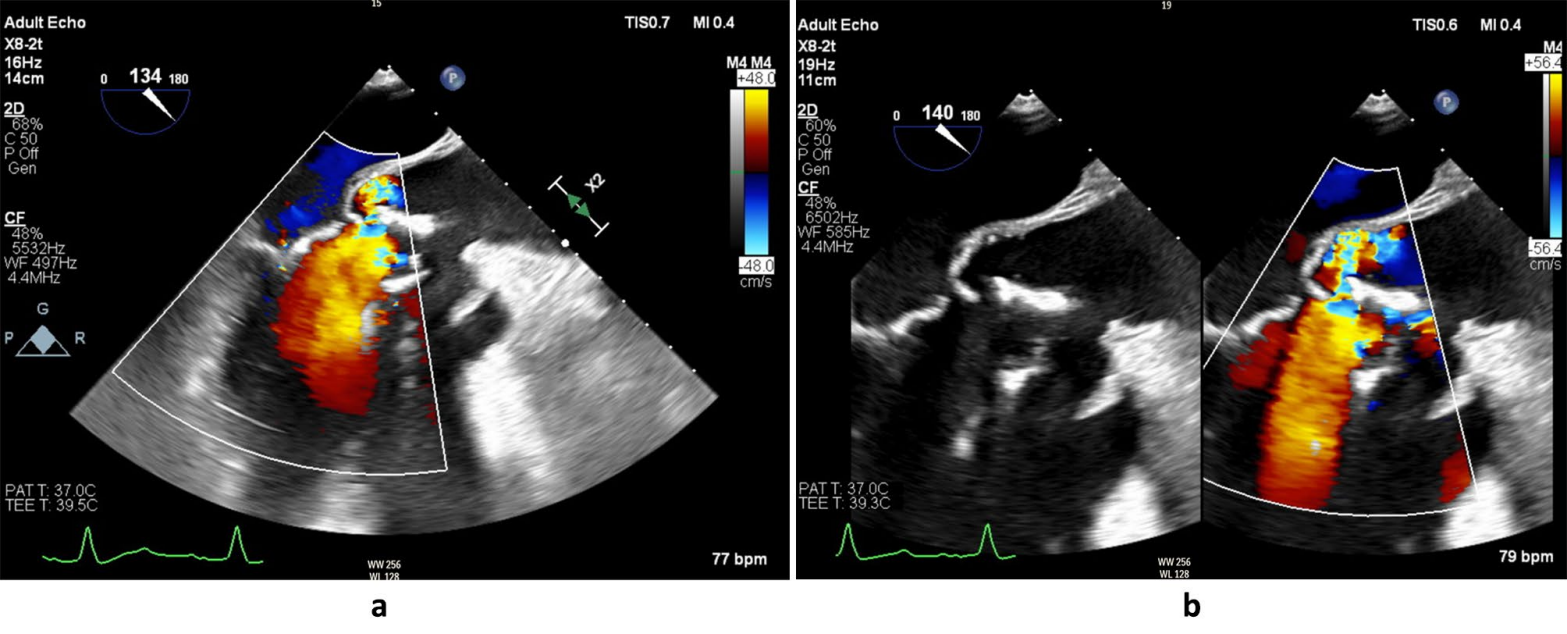
**a** & **b**: Transoesophageal echocardiogram long axis view of the dilated sinus of valsalva, aortic regurgitation and rocking of the aortic valve

**Fig. 3:**
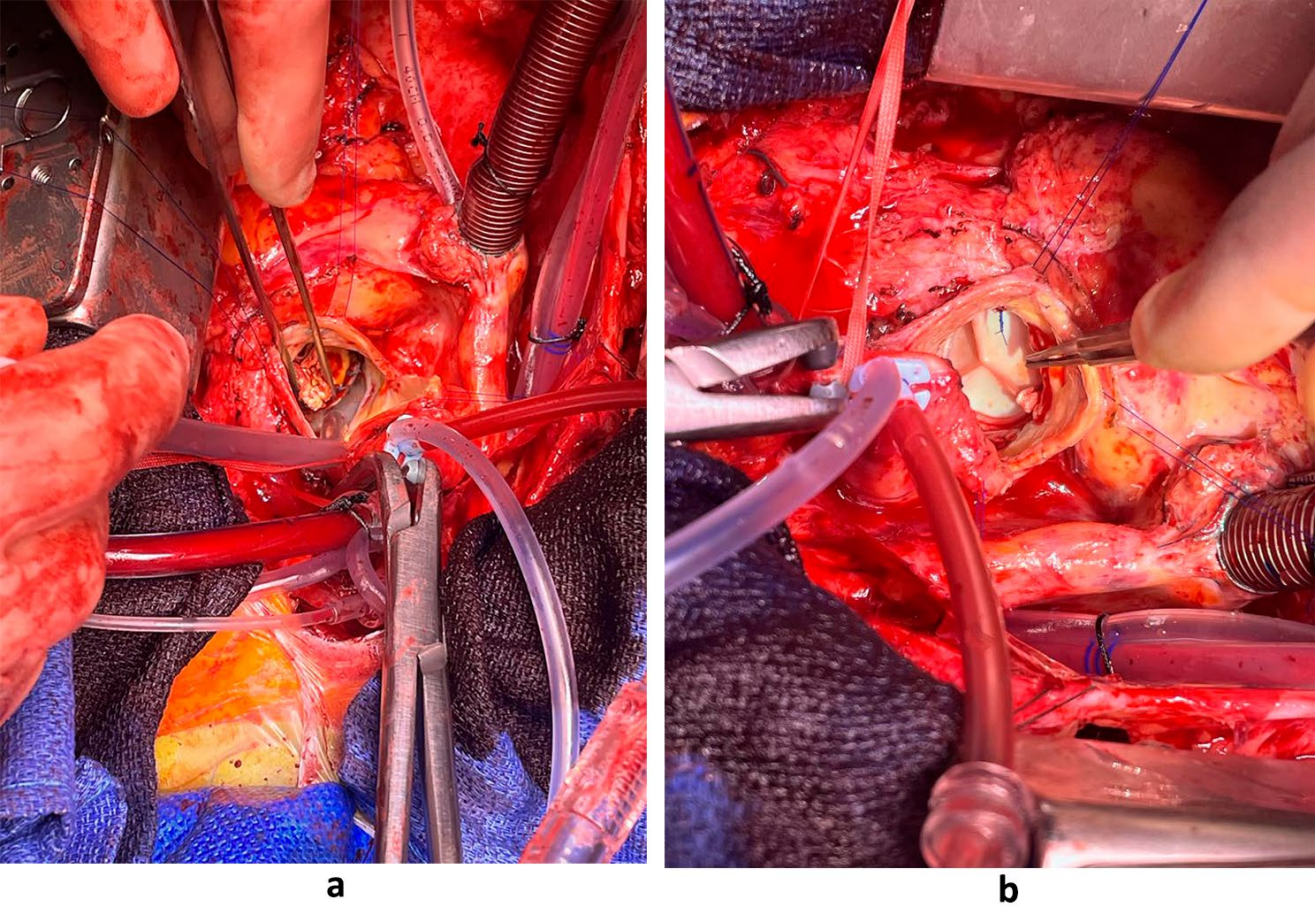
**a**: Valve from 2013 in the process of being explanted. **b**: New valve from redo surgery

Intraoperatively, the previous bioprosthetic valve was held in place by only three sutures whilst the rest of the valve was completely dehisced and rocking (Fig. 2a & b). The valve had degenerated with severe stenosis but was functioning as a flap valve with a hinge. A large multilobulated pseudoaneurysm with the inferior margin abutting the annulus and measuring 4-5 cm vertically and extending circumferentially up to 90% of the aortic root, whilst sparing a small area above non-coronary and right coronary commissure. The coronary ostia were superiorly displaced, and there was anomalous origin of left circumflex artery (LCx) arising from right coronary artery (RCA), having a retro-aortic course. Intraoperative imaging demonstrated the diseased aortic valve and the new aortic valve can be seen in Fig. 3a and b.

In view of the complex coronary origin and course in relation to the pseudoaneurysm, it was decided to perform a sub-coronary root replacement as there was a clear margin of 1-1.5 cm above the superior rim of the pseudoaneurysm. A composite valve conduit (23 mm Perimount Magna Ease bovine pericardial aortic valve (Edward Lifesciences, CA, USA) with 28 mm Gelweave (Terumo Aortic, UK) straight tube graft) was sutured to the aortic annulus and the superior rim of the pseudoaneurysm thereby excluding the pseudoaneurysm from the root and circulation.

Transoesophageal echocardiography (TOE) performed intraoperatively and transthoracic echocardiography (TTE) done postoperatively confirmed a well seated aortic valve and obliteration of the pseudoaneurysm. Heart valve tissue was sent intraoperatively and returned back culture negative. The patient had an uneventful post operative course and was discharge a five days later to home from hospital.

## Discussion

Since 1956, replacing the aortic valve and root has been an evolving technique, with the standard surgical management involving a replacing the entire aortic valve with a composite valve graft and reimplantation of the coronary ostia [1]. Aortic root replacement is a surgical procedure that involves replacing the aortic root with a prosthetic graft while preserving the native aortic valve. It is a viable option for patients who present with isolated aortic root disease or minimal aortic valve pathology [2]. Despite the varied technique in how we perform aortic root surgery, it has always been necessary to mobilise and reimplant the coronaries. In endocarditis of prosthetic valve, usually homograft is used for redo aortic root replacement [3, 4]. Prosthetic valve infective endocarditis constitutes 1-6% of all prosthetic valve infective endocarditis [5]. Prosthetic valve endocarditis rarely occurs after aortic valve replacement, and the concurrent presence of aortic root pseudoaneurysm further complicate the clinical presentation [6].

Treatment should involve a multidisciplinary team approach. Various techniques have been employed to manage aortic root pseudoaneurysm in view of prosthetic valve dehiscence. In a study by Pereda et al., they isolated the coronaries, skeletonised the aortic root as done during valve-sparing procedures, separating the aorta from its surrounding tissues. Pledgeted sutures are then passed through the full thickness of the aorta from outside the aortic wall to inside [7]. In another technique, Hadjinikolaou et al. found success in a technique involving excising most of the aortic root wall except the coronary ostia. They reinforced the remaining aortic root wall with external Dacron and used sutures and a pericardial patch for the posterior aortic wall anastomosis. Additionally, they suggested possibly considering such surgical technique in the elderly where higher morbidity and mortality rates can be expected in patients requiring aortic root replacement [8]. 

In addition to this patient being elderly with aortic root pseudoaneurysm, he was also in the 1% of patients who have coronary artery anomalies from the aorta, the most common anomaly being the anomalous left circumflex artery (LCX) from the right coronary artery (RCA). Presence of complex coronary artery anatomy increases the complexity of the procedure, since there is high risk of compromising one of the common ostium on transferring or reimplantation. These patients are at high risk for suffering coronary vessel injury, LCX injury is possible during resection or suturing and, incorrect sizing of graft or prosthesis resulting in coronary artery distortion. A study by Leibrich et al. described an alternative technique for aortic root replacement that avoided the need for coronary ostial mobilization or reimplantation [9]. But this technique involves the use of a specially designed aortic root prosthesis, which is shaped to fit the patient’s individual anatomy and maintain the integrity of the coronary ostia. In addition, none of the reported 29 cases consisted of redo aortic procedure.

To date, despite extensive literature search, there appears to be no case report or case series that discusses a case like ours. The nature of the complex anatomy and the fact that the case was an urgent redo, highlights that such a technique can be performed and should be considered in and more complex patients.

## Conclusion

In conclusion, sub-coronary aortic root replacement may be a viable option to be considered when operating on elderly patients and patients with complex coronary anatomy and prosthetic valve dehiscence. A multidisciplinary approach is necessary for the treatment of prosthetic valve dehiscence, including antibiotics, surgical intervention, and close follow-up.

## Data Availability

Not applicable.
